# Manual compression and reflex syncope in native renal biopsy

**DOI:** 10.1007/s10157-018-1560-8

**Published:** 2018-03-14

**Authors:** Yoichi Takeuchi, Yoshie Ojima, Saeko Kagaya, Satoshi Aoki, Tasuku Nagasawa

**Affiliations:** Division of Nephrology, Department of Medicine, Japanese Red Cross Ishinomaki Hospital, Nishimichishita-71 Hebita, Ishinomaki, Miyagi 986-8522 Japan

**Keywords:** Percutaneous renal biopsy, Complications, Standard practice, Quality of life, Logistic regression model

## Abstract

**Background:**

Complications associated with diagnostic native percutaneous renal biopsy (PRB) must be minimized. While life threatening major complications has been extensively investigated, there is little discussion regarding minor bleeding complications, such as a transient hypotension, which directly affect patients’ quality of life. There is also little evidence supporting the need for conventional manual compression following PRB. Therefore, this study evaluated the relationship between minor and major complications incidence in patients following PRB with or without compression.

**Methods:**

This single-center, retrospective study included 456 patients (compression group: *n* = 71; observation group: *n* = 385). The compression group completed 15 min of manual compression and 4 h of subsequent strict bed rest with abdominal bandage. The observation group completed 2 h of strict bed rest only. The primary outcome of interest was transient symptomatic hypotension (minor event).

**Results:**

Of the 456 patients, 26 patients encountered intraoperative and postoperative transient hypotension, which were considered reflex syncope without tachycardia. Univariate analysis showed that symptomatic transient hypotension was significantly associated with compression. This association remained significant, even after adjustment of covariates using multivariate logistic regression analysis (adjusted odds ratio 3.27; 95% confidential interval 1.36–7.82; *P* = 0.0078).

**Conclusion:**

Manual compression and abdominal bandage significantly increased the frequency of reflex syncope during native PRB. It is necessary to consider the potential benefit and risk of compression maneuvers for each patient undergoing this procedure.

**Electronic supplementary material:**

The online version of this article (10.1007/s10157-018-1560-8) contains supplementary material, which is available to authorized users.

## Introduction

Ultrasound-guided renal biopsy is a very useful tool for the diagnosis of kidney diseases. Meanwhile, the risk of complications from the procedure must be minimized. Major complications associated with native percutaneous renal biopsy (PRB) are often defined as those resulting in the need for interventions, such as blood transfusion and radiological or surgical intervention [1]. In this regard, many clinicians have examined the preoperative risks of major bleeding complications in detail [1–4]. On the other hand, minor events are partly defined as those resulting in gross hematuria and symptomatic perinephric hematoma that resolve without further intervention [2]. A minor event is likely neglected because it is not life-threatening. Additionally, despite their transient manner, back pain and symptomatic hypotension that are associated with PRB impair the quality of life (QOL) of hospitalized patients. However, detailed investigation on these minor events remains unclear.

Guidelines for standard practice of PRB are currently being developed in each country [5, 6]. In spite of the fact that the potential risk of complications should be assessed for every step of the PRB procedure, there is little evidence on the usefulness of manual compression for PRB, and the compression technique is different at each facility. Compression technique and enforced bed rest are likely to influence patients’ psychological burden (such as lumbago) [7], and increase the risk of developing deep vein thrombosis or pulmonary embolism [8].

We empirically noticed transient hypotension during manual compression in PRB, and questioned the usefulness of manual compression. As a result, we decided to investigate whether manual compression and enforced bed rest during PRB affected the incidence of symptomatic transient hypotension. Additionally, we determined the clinical effectiveness of manual compression for PRB by comparing the presence of compression with the occurrence of minor and major complications.

## Materials and methods

### Study design and patient population

This retrospective observational study was conducted in 456 consecutive patients who underwent PRB from September 2013 to April 2017 in the Department of Nephrology at the Japanese Red Cross Ishinomaki Hospital. The indication and contraindication criteria of PRB were based on our previous study [4].

### Outcome measures and definitions

The patient’s age, gender, body mass index, early morning blood pressure (BP) on the day of PRB, serological clinical data with urinalysis during admission for PRB, and the use of antithrombotic drugs were investigated. Postoperative measures included gross hematuria and presence of transient hypotension or syncope, hemoglobin (Hgb) value 1 day after PRB, use of blood transfusion, indication of angiographic intervention or bladder obstruction, bleeding volume, and kidney volume.

The severity of the complication was categorized as minor or major. Minor complications were defined as those resulting in gross hematuria and symptomatic hypotension, but spontaneously resolved without further intervention. Transient hypotension, which was the primary outcome in this study, was defined as a decrease of 20 mmHg or more during the procedure, in addition to related symptoms, such as nausea, vomiting, abdominal discomfort, or excessive perspiration. Within 48 h of hospitalization, cases that presented with hypotension after returning to the hospital ward and those whose transient loss of consciousness was clearly noticeable were also included. Major complications were those resulting in the need for intervention, such as a transfusion of blood products or invasive procedure (angiographic or surgical), those resulting in acute renal obstruction or failure or septicemia, or those resulting in death. When assessing the frequency of blood transfusions, we only included biopsy-related transfusions. We excluded irrelevant cases of transfusion that were obviously due to other disorders (e.g., gastrointestinal bleeding or preparation for surgery) or slowly-progressive anemia (e.g., transfusion for rapidly progressive glomerulonephritis). Conversely, transfusions that were completed within 3 days after the biopsy were included as biopsy-related transfusions, even if the description was not clearly found on the medical record. These transfusions were included because implementation of blood transfusion usually depends on the judgment of the on-site performer, and we were concerned of underestimating the frequency of major complications, considering the retrospective nature of this study.

We used the formula recommended by the Japanese Society of Nephrology for estimated glomerular filtration ratio (eGFR) [9]. Bleeding volume and kidney volume were quantitatively measured by a radiologist using imaging software (Ziostation 2; Ziosoft, Inc., Tokyo, Japan) from the abdominal computed tomography performed on the day after PRB, as shown in detail in our previous study [4].

### Percutaneous renal biopsy protocol

Informed written consent for the procedure was obtained from all patients. Patients were generally hospitalized for 4 days, based on the clinical practice pathway for PRB. Medical history for infections and complete blood count, clinical chemistry, and clotting parameters were determined prior to the procedure. Among the antithrombotic agents, aspirin was withdrawn 3 days before hospitalization, and clopidogrel and ticlopidine were withdrawn 4 days before hospitalization. Upon admission, warfarin sodium was heparinized, and the drug was then withdrawn 5 days prior to PRB; the intravenous heparin was eventually discontinued 4 h before the biopsy procedure. The biopsy started at 10:00 am without breakfast. With a sterile condition and a prone posture, the biopsy was performed by nephrologist fellows in the treatment room under a controlled temperature of 24 °C. Under ultrasonographic guidance (HI VISION Avius®; HITACHI Medical Corp., Tokyo, Japan), subcutaneous tissue and fascia were locally anesthetized. If the systolic BP was more than 160 mmHg before puncture, the calcium channel blocker nicardipine was intravenously administered at a concentration of 0.1 mg/dL until the systolic BP was lowered to 160 mmHg or less. Three core biopsies were obtained by puncturing the lower pole of the kidney using an automated, spring-loaded biopsy device and a 16-cm, 16-gauge needle (Tru-Core II; Angiotech Pharmaceuticals, Inc., Gainesville, FL, USA). BP was automatically measured using a monitor unit (DS-7110; Fukuda Denshi, Nagoya, Japan) every 5 min during PRB. The procedures were performed according to previously reported recommendations [10].

After the procedure, the patients who underwent PRB up to November 2014 received manual compression, while those who underwent PRB since November 2014 received neither manual compression nor abdominal bandage. Patients who received manual compression for 15 min (compression group) had a 5-pound abdominal compression bandage placed over the biopsy site. Then, the nurses rolled the patient into the supine position and prescribed strict bed rest for 4 h in the hospital ward. Conversely, the patients who did not receive manual compression (observation group) independently changed their posture to the supine position, and were prescribed strict bed rest for 2 h. We instructed all patients to rest on the bed for an additional 6 h or more. Nurses closely observed the patients in the hospital ward. Patients that presented with hypotension or syncope were administered accelerated external liquid instillation on site, and BPs were normalized using intravenous atropine sulfate. Eventually, we confirmed that accompanying symptoms of the patients were relieved.

### Statistical analysis

Results are reported as mean ± standard deviation for continuous values with a normal distribution, or median [25th and 75th percentiles] for the values that were not normally distributed. Categorical values are reported as integers, frequencies, and percentages. Normality was assessed using the Shapiro–Wilk test. In the univariate analyses, *t* test was used for parametric variables, and Mann–Whitney *U* test was used for non-parametric variables. Fisher’s exact test was used for categorical variables. Logistic regression analysis was used to control for the possible confounding variables related to bleeding complications. Adjustments were made for the following variables: age, sex, body mass index, early morning systolic BP, antithrombotic drug, eGFR, pre-PRB Hgb, and platelet count. Risk estimates were noted as unadjusted and adjusted odds ratio and 95% confidence intervals. All statistical data were analyzed at a significant level of 0.05 using EZR (Saitama Medical Center, Jichi Medical University, Saitama, Japan), which is a graphical user interface for R version 3.2.1 (The R Foundation for Statistical Computing, Vienna, Austria) [11].

## Results

Over a 4-year period, 456 patients underwent PRB at the Japanese Red Cross Ishinomaki Hospital. Of the 456 patients, 71 patients were in the compression group and 385 in the observation group. Clinical characteristics of the patients are shown in Table 1. There was no difference between groups for age, body mass index, early morning BP, the use of antithrombotic drugs, kidney volume, and urine protein. Platelet count was significantly lower in the observation group than the compression group.

**Table 1:** Clinical background of patients by group

	Observation (*N* = 385)	Compression (*N* = 71)	*P* value
Age (year)	66.0 [54.0, 76.0]	64.0 [49.0, 73.0]	0.24
Sex, women	144 (37.4)	28 (39.4)	0.79
BMI (kg/m^2^)	24.6 [22.3, 27.4]	24.2 [22.1, 27.0]	0.51
Systolic BP (mmHg)	136.0 [121.0, 154.0]	134.0 [124.0, 147.0]	0.55
Diastolic BP (mmHg)	76.0 [68.0, 85.0]	77.0 [69.0, 87.5]	0.33
Antithrombotics usage	73 (19.1)	16 (22.5)	0.52
Platelet count (10^4^/µL)	22.7 [18.1, 28.9]	25.7 [21.3, 32.0]	0.0081*
PT (%)	106.0 [93.0, 122.0]	102.5 [89.5, 111.5]	0.10
aPTT (s)	30.5 [27.6, 34.0]	29.3 [27.1, 33.7]	0.42
Creatinine (mg/dL)	1.49 [1.01, 2.13]	1.30 [0.89, 2.00]	0.07
eGFR (mL/min/1.73 m^2^)	27.9 [19.0, 44.0]	33.0 [20.2, 53.2]	0.06
Pre-PRB Hgb (g/dL)	12.4 [10.7, 13.8]	12.5 [10.3, 14.3]	0.38
Kidney volume (mL)	159.1 [127.3, 194.1]	159.2 [135.7, 189.2]	0.72
Urine protein (g/gCre)	1.20 [0.40, 3.65]	1.60 [0.45, 3.45]	0.56

Continuous variables are presented as median [25th and 75th percentile]. Among the continuous variables, *t* test was used for parametric variables and Mann–Whitney *U* test for non-parametric variables. Categorical variables are presented as number (percentage). Fisher’s exact test was used for categorical variables

*BMI* body mass index, *BP* blood pressure, *PT* prothrombin time, *aPTT* activated partial thromboplastin time, *eGFR* estimated glomerular filtration rate, *PRB* percutaneous renal biopsy, *Hgb* hemoglobin

*Significant difference at *P* < 0.05

**Fig. 1:**
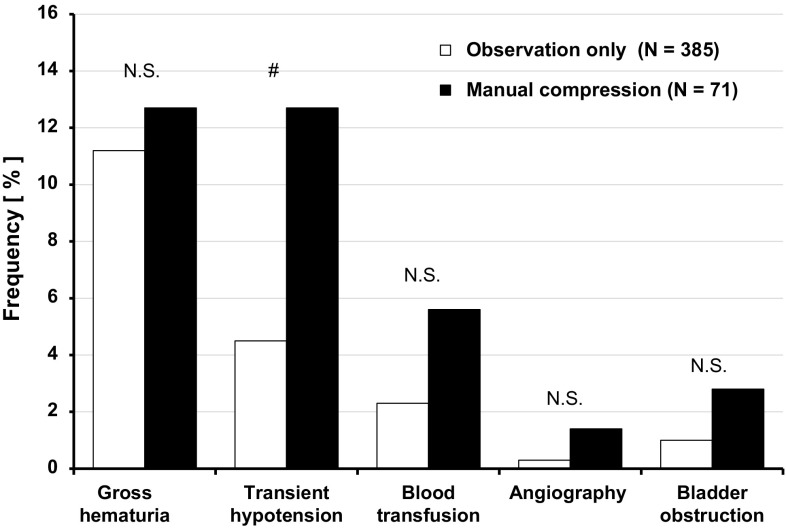
Bleeding complications by group. Gross hematuria and transient hypotension were evaluated as minor complications, while blood transfusion usage, angiographic intervention, and bladder obstruction were assessed as major complications. Fisher’s exact test was used for each categorical variable to compare differences between the compression and observation groups. *Significant difference at *P* < 0.05. *PRB* percutaneous renal biopsy, *Hgb* hemoglobin

**Fig. 2:**
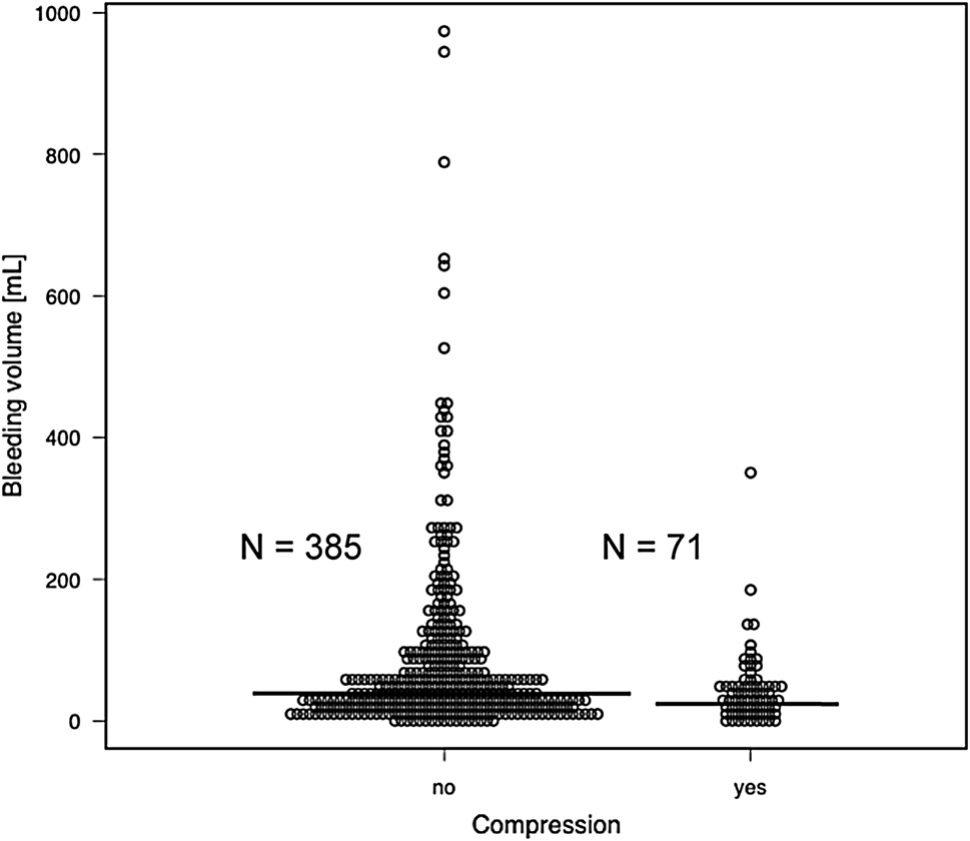
Distribution of bleeding volume during the percutaneous renal biopsy. The dot plot displays the bleeding volume of 456 patients who underwent percutaneous renal biopsy in this study. Mann–Whitney *U* test was used for this non-parametric variable. The median values are represented as solid lines. The difference of the median values between groups was about 15 mL, and was significantly greater in the observation group than in the compression group (*P* < 0.01)

The frequencies of minor and major complications by group are shown in Fig. 1. Gross hematuria and transient hypotension were evaluated as minor complications. Among the minor complications in this study (16.7%, 76/456 patients), there was no difference in the incidence of gross hematuria after PRB between groups; however, transient hypotension or syncope was significantly greater in the compression group than in the observation group. In contrast, the use of blood transfusion, presence of hemodynamic condition requiring angiographic or surgical intervention, and bladder obstruction were assessed as major complications. Major complications were observed in 18/456 (3.9%) patients. Of note, no significant difference was observed for each of the major complications between groups. Moreover, no events including surgical interventions, septicemia, or death were observed in any patients. In addition, we plotted the distribution of bleeding volume (Fig. 2), and compared the change in Hgb concentrations before and after the procedure between the groups, as shown in the supplementary table (Online Resource 1). There was no significant difference for the absolute Hgb value between the two groups. As for the bleeding volume, the difference of the median values between groups was about 15 mL, and the observation group had a significantly greater volume than the compression group (44.7 mL [21.0, 97.4] in the observation group vs. 31.1 mL [15.2, 52.2] in the compression group; *P* < 0.01).

Of 456 patients, 26 patients (compression group, *n* = 9; observation group, *n* = 17) developed transient hypotension (Table 2**)**. In the compression group, most of the symptomatic hypotension occurred during the manual compression in the treatment room. The most frequent symptom was perspiration. Over 80% of patients who developed transient hypotension did not have pain at the puncture site. Of importance, there were no cases with concomitant tachycardia when being monitored for the reduction of BP. Additionally, patients who were prescribed alpha adrenergic antagonists were in the observation group only. Further, intravenous nicardipine for optimizing BP prior to PRB was administered in only two patients from the compression group.

**Table 2:** Characteristics of transient hypotension in patients by group

	Total (*N* = 26)	Observation (*N* = 17)	Compression (*N* = 9)	*P* value
Location at onset				
Treatment room	16 (61.5)	8 (47.1)	8 (88.9)	0.09
Hospital ward	10 (38.4)	9 (52.9)	1 (11.1)	
Symptoms				
Perspiration	11 (42.3)	8 (47.1)	3 (33.3)	
Nausea and/or vomiting	9 (34.6)	7 (41.2)	2 (22.2)	
Uncomfortable sensation	9 (34.6)	8 (47.1)	1 (11.1)	
Pain at puncture site	5 (19.2)	3 (17.6)	2 (22.2)	
Dizziness	4 (15.4)	4 (23.5)	0 (0.0)	
Administration of alpha adrenergic antagonist	2 (7.7)	2 (11.8)	0 (0.0)	0.53
Use of intravenous nicardipine	7 (26.9)	5 (29.4)	2 (22.2)	1.00
Decline of systolic BP (mmHg)		52.6 ± 18.4	41.9 ± 25.5	0.24
HR at onset (bpm)		60.7 ± 15.3	66.5 ± 16.3	0.41

Data are presented as number, number (percentage), or mean ± standard deviation

*BP* blood pressure, *HR* heart rate

**Fig. 3:**
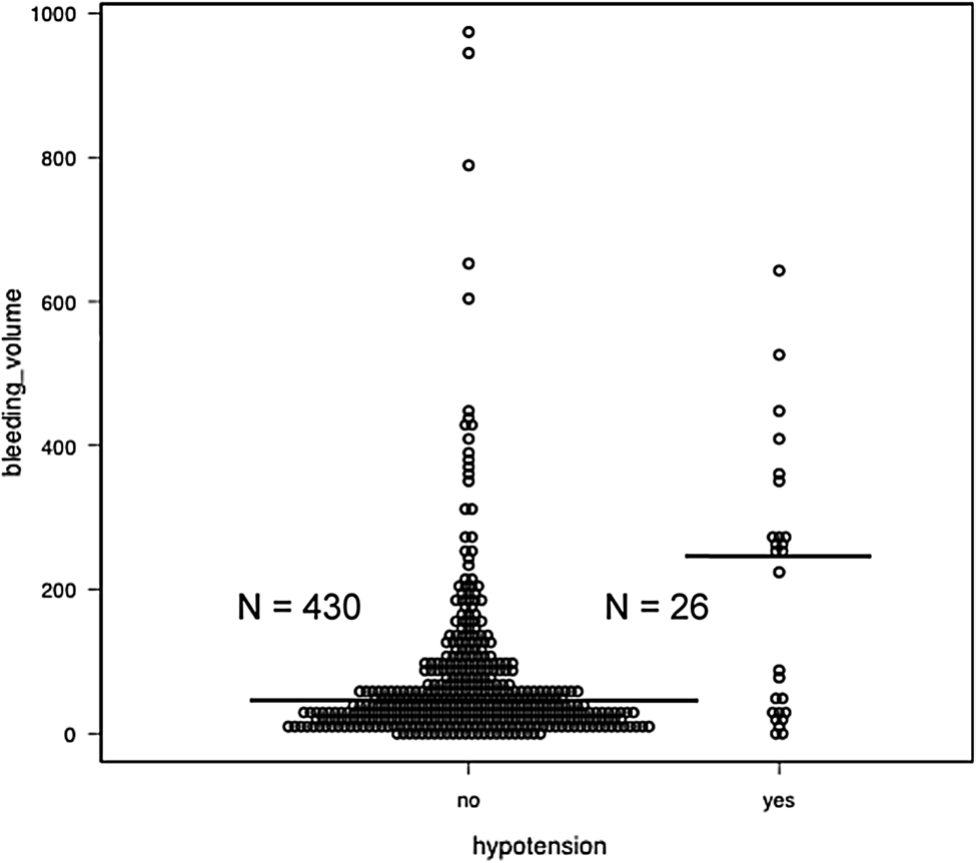
Comparison of bleeding volumes between patients with and without transient hypotension. The dot plot illustrates the bleeding volume of 456 patients with and without hypotension. The median values are represented as solid lines. Mann–Whitney *U* test was used for this non-parametric variable

To know whether the transient hypotension depended on bleeding volume, we compared the actual amount of bleeding among the patients with or without hypotension. There represented a clear significance (median of 38 mL in asymptomatic patients vs. median of 237 mL in patients developing hypotension; *P* = 0.0012 using the Mann–Whitney *U* test) that is shown in the Fig. 3. However, the bleeding volume in the patients developing transient hypotension was widely distributed. Next, we examined the occurrence of hypotension and major bleeding complication by dividing hematoma volume into two groups. In the group with massive bleeding over 400 mL, the frequency of transient hypotension was significantly higher than those in the group of 400 mL or less. However, no significance was observed in the occurrence of major complications between the groups (Online Resource 2).

Unadjusted and adjusted estimates of the association between different risk factors and symptomatic transient hypotension are presented in Table 3. All of the included covariates were identified by previous studies as confounding factors for preoperative risk of bleeding complications [3, 5, 12, 13]. Symptomatic transient hypotension after PRB was significantly associated with the presence of manual compression in the univariate analysis. When using the logistic regression model, the significant association between symptomatic transient hypotension and compression remained significant, even after adjustment for the other eight risk factors (adjusted odds ratio 3.27; 95% confidential interval 1.36–7.82; *P* = 0.0078).

**Table 3:** Logistic regression analysis assessing the risks for symptomatic transient hypotension

Risk factors	Unit of increase	Unadjusted OR (95% CI)	*P* value	Adjusted OR (95% CI)	*P* value
Age (years)	10	0.93 (0.74–1.16)	0.51	0.90 (0.67–1.20)	0.48
Sex, male	NA	0.82 (0.37–1.82)	0.62	0.91 (0.37–2.25)	0.84
BMI (kg/m^2^)	1	0.96 (0.87–1.06)	0.40	0.98 (0.88–1.08)	0.63
Systolic BP (mmHg)	10	0.86 (0.71–1.04)	0.11	0.85 (0.68–1.05)	0.12
Antithrombotics usage	NA	0.74 (0.25–2.20)	0.59	0.74 (0.24–2.34)	0.61
eGFR (mL/min/1.73 m^2^)	10	1.04 (0.89–1.21)	0.64	0.97 (0.79–1.19)	0.74
Pre-PRB Hgb (g/dL)	1	0.97 (0.82–1.15)	0.74	0.93 (0.76–1.15)	0.52
Platelet count (10^4^/µL)	10^5^	1.00 (0.96–1.04)	0.93	0.99 (0.95–1.03)	0.62
Compression	NA	3.12 (1.33–7.30)	0.0089*	3.27 (1.36–7.82)	0.0078*

*OR* odds ratio, *CI* confidence interval, *BMI* body mass index, *BP* blood pressure, *eGFR* estimated glomerular filtration rate, *PRB* percutaneous renal biopsy, *Hgb* hemoglobin

*Significance at *P* < 0.05

## Discussion

This was an observational study that retrospectively examined the effectiveness of compression maneuver following ultrasound-guided native PRB. Compression on the puncture site after PRB lowered the bleeding volume by 15 mL compared to the observation group. However, the use of manual compression did not reduce the occurrence of major events compared to non-compression. Furthermore, logistic regression analysis revealed that the use of manual compression increased the risk of symptomatic transient hypotension by approximately three times, even after adjustment for other known risk factors.

There was no difference in the rate of occurrence of the major complications between the compression group and the observation group. The major complications of PRB have been extensively examined. Major events occur in 0–6% of patients during PRB [3], and the following predictors have been identified: coagulopathy; kidney dysfunction (serum creatinine more than 2.0 mg/dL); low Hgb concentration; use of a wide needle (i.e., 14-gauge); women; and older age [12]. The incidence rates of major complications in the present study were in good agreement with previous reports [3]. In this study, the difference in the amount of bleeding volume with or without compression was only 15 mL. Previous studies reported that bleeding complications could not be predicted from the hematoma size that was measured using ultrasound and Hgb reduction rate [2, 14]; thus, we suggest that the small reduction of bleeding volume, which was expected from compression maneuver, likely did not contribute to the prevention of complications. Therefore, in an effort to decrease the occurrence of major events, it is likely safe to not compress the puncture site and to not use abdominal bandage.

Here, the result that manual compression increased the frequency of symptomatic hypotension must be evaluated. The symptomatic transient hypotension observed in this study was classified as a reflex syncope (also known as neurally mediated syncope), evidenced by no patients experiencing concomitant tachycardia following the minor event. Reflex syncope is generally triggered by persistent and unpleasant stimuli, and is accompanied with spontaneous bradycardia and vasodilation. Risk factors underlying reflex syncope include psychiatric diseases (such as depression), history of heart diseases, or drug usage (such as alpha adrenergic antagonist). Clinical findings include the autonomic symptoms, such as headache, dizziness, nausea, and perspiration. During the PRB procedure, it seems that unpleasant situations, such as pain and anxiety, triggered the reflex syncope. Therefore, by completing the 15 min of manual compression, the patients in the compression group had to endure additional discomfort, which potentially impaired the patients’ QOL. However, it was difficult to quantify the extent of individual manual pressure. Among the patients who had symptomatic hypotension in this study, there were no recipients who used alpha adrenergic antagonists, and few patients who used intravenous nicardipine from the compression group. Thus, the contribution of the medications to reflex syncope seems minimal. Further studies with a larger compression group sample size would be suitable to analyze these effects.

We are interested in the association between manual compression and transient hypotension in this study. Although we obtained a result that the transient hypotension occurred higher in the group of retroperitoneal bleeding 400 mL or more in Online Resource 2, it is not surprising that the massive bleeding posed an episode of hypotension. What we should focus on are the cases who suffer BP reduction despite a small bleeding volume, and the fact that most of the hypotension emerged just at the time of compression procedure. No significant difference was observed in the occurrence of major complication between the group with bleeding volume of 400 mL or more and those with bleeding volume of 400 mL or less. It is also noteworthy that major complications can not simply be predicted with only an amount of retroperitoneal bleeding, as described above [2, 14]. But, it can not be discussed further for inadequacy of its sample size.

There are several strengths in the present study that warrant attention. First, this study focused on reflex syncope, one of the minor events that directly affected the patient’s QOL following PRB. To date, detailed investigations on minor events after PRB are limited. However, since further interventions for minor complications are deemed unnecessary, most clinicians are not motivated to investigate minor complications, such as small hematoma and back pain. Furthermore, since the minor complications are temporary and spontaneous in nature, and are often difficult to identify, the incidence of missing data tends to increase, which is especially problematic for retrospective observational research. However, although reflex syncope after PRB manifests temporary, it presents with obvious symptoms; therefore, reflex syncope can be easily detected with careful monitoring after PRB. As such, we consider reflex syncope as one of the most reliable outcome measures among the minor complications.

Second, the content of this clinical study is feasible and can be conducted well at any hospital. There were no special skills necessary for medical personnel to complete the practical protocols and monitoring techniques used in this study. Since the study population included a continuous cohort from a single facility using a clinical pathway, it was possible to uniformly evaluate the technical competency between the performers (those who completed the biopsy), and to reduce the missing value of a series of measurement items from the nurses. By frequently measuring vital signs during the procedure, highly reliable clinical research could be conducted without omitting transient hypotension, when possible.

It is important to note the clinical and social significance of this study. In review articles, some researchers reported uncertainties regarding hemostasis by compression itself, and advocated for the need of observational studies for this aspect [3, 5]. While confirmation of the compression technique’s usefulness is necessary, we also need to pay attention to its potential harm. For example, Ishikawa and colleagues focused on evaluating one of the conventional steps of PRB. The researchers determined that shortening the duration of strict bed rest to 2 h had no effect on increased bleeding risk, but it also improved the incidence of back pain [7]. With the concern of major complications in our study, we prescribed a total bed rest of about 8 h or more. Accordingly, we showed that compression technique and 4-h strict bed rest slightly reduced bleeding volume, but also increased the risk of symptomatic transient hypotension by approximately three times. Therefore, with regards to bleeding complications, we should consider that the actual bleeding volume from PRB does not necessarily relate with the frequency of minor complications.

Recently, guidelines for standard practice of PRB are being developed in each country, and it is necessary to gather extensive clinical evidence [5, 6]. In establishing the guidelines for successful PRB practice, strategies to minimize minor events should be considered in an effort to improve the patient’s QOL. Atwell et al. emphasized that the most common clinical indicators of bleeding in ultrasound-guided biopsy are pain and hemodynamic instability [15]. Since serious sequelae are continuously induced from minor bleeding complications, it is important to promptly handle the minor events to reduce the incidence of subsequent major events. A previous report on direct compression using ultrasound probes in pediatric patients presenting color Doppler signal after procedure determined that compression was effective in reducing bleeding volume in the children, whose small muscle volume easily conducted the pressure [16]. Therefore, manual compression and abdominal bandage should be indicated for children who have a risk of bleeding complications to reduce the bleeding volume after PRB.

There are limitations in this study. We are concerned that this study had much more cases of blood transfusion than those of previous studies reported from Japan (9 of 385 patients in observation group vs. 4 of 71 patients in compression group, in Fig. 1). On the grounds that transfusion (as a major complication) should not be underestimated, we had no choice but to include transfusion cases of unknown purpose (due to lack of information) aside from clearly identified biopsy-related transfusions. This is one of the reasons for increasing the total number of blood transfusions. Incomplete case capture is a limit of a retrospective study design; therefore, fewer cases of true biopsy-related transfusions would be estimated in this study. Additionally, it was impossible to randomly assign the patients to the groups, because patients received compression during the first half of the entry period (from 2013 to 2014) and patients did not receive compression during the second half of the entry period (from 2014 to 2017). We compared two groups with different periods of PRB completion because our facility decided to discontinue manual compression after we noticed an increase of undesired symptomatic hypotension. And, the practice in these two groups was quite different in the time of bed rest from those recommended by Japanese Society of Nephrology guideline [10]. Here, we could not discuss the question that different bed-rest time between groups influenced the bleeding complications and bleeding volume after PRB. Additionally, we could not discuss the risks of back pain and deep vein thrombosis or pulmonary embolism due to pressure from the compression and the enforced 4-h bed rest, because this observational study did not collect the clinical information. At last, the sample size of this study was not large enough to analyze other potential confounding factors affecting this primary outcome.

In summary, manual compression technique did not change the occurrence of major events compared to non-compression. However, the use of compression increased the risk of symptomatic transient hypotension by approximately three times. We should pay careful attention to the benefit from manual compression as well as potential risk for hypotension.

## Electronic supplementary material

Below is the link to the electronic supplementary material.

Supplementary material 1 Evaluation measures for hemorrhage. Continuous variables are presented as median [25th and 75th percentile]. Mann-Whitney *U* test were used for these non-parametric variables. * significant difference at *P* < 0.05. PRB, percutaneous renal biopsy; Hgb, hemoglobin (PPTX 71 KB)

Supplementary material 2 Comparison of complications by hemorrhage. Data are presented as number (percentage). Major complication was assessed by counting the case of blood transfusion usage, angiographic intervention, or bladder obstruction. Fisher’s exact test was used for each binominal variable to compare differences between two groups (DOCX 15 KB)
