# Supplementary material for: Manual compression and reflex syncope in native renal biopsy
Source: Clin Exp Nephrol. 2018 Mar 14;22(5):1100–7. doi: 10.1007/s10157-018-1560-8 (PMC6154117; doi:10.1007/s10157-018-1560-8)
Supplement: Supplementary file 1 — Supplementary material 1 Evaluation measures for hemorrhage. Continuous variables are presented as median [25th and 75th percentile]. Mann-Whitney U test were used for these non-parametric variables. * significant difference at P < 0.05. PRB, percutaneous renal biopsy; Hgb, hemoglobin (PPTX 71 KB) [file 10157_2018_1560_MOESM1_ESM.pptx]

## Slide 1
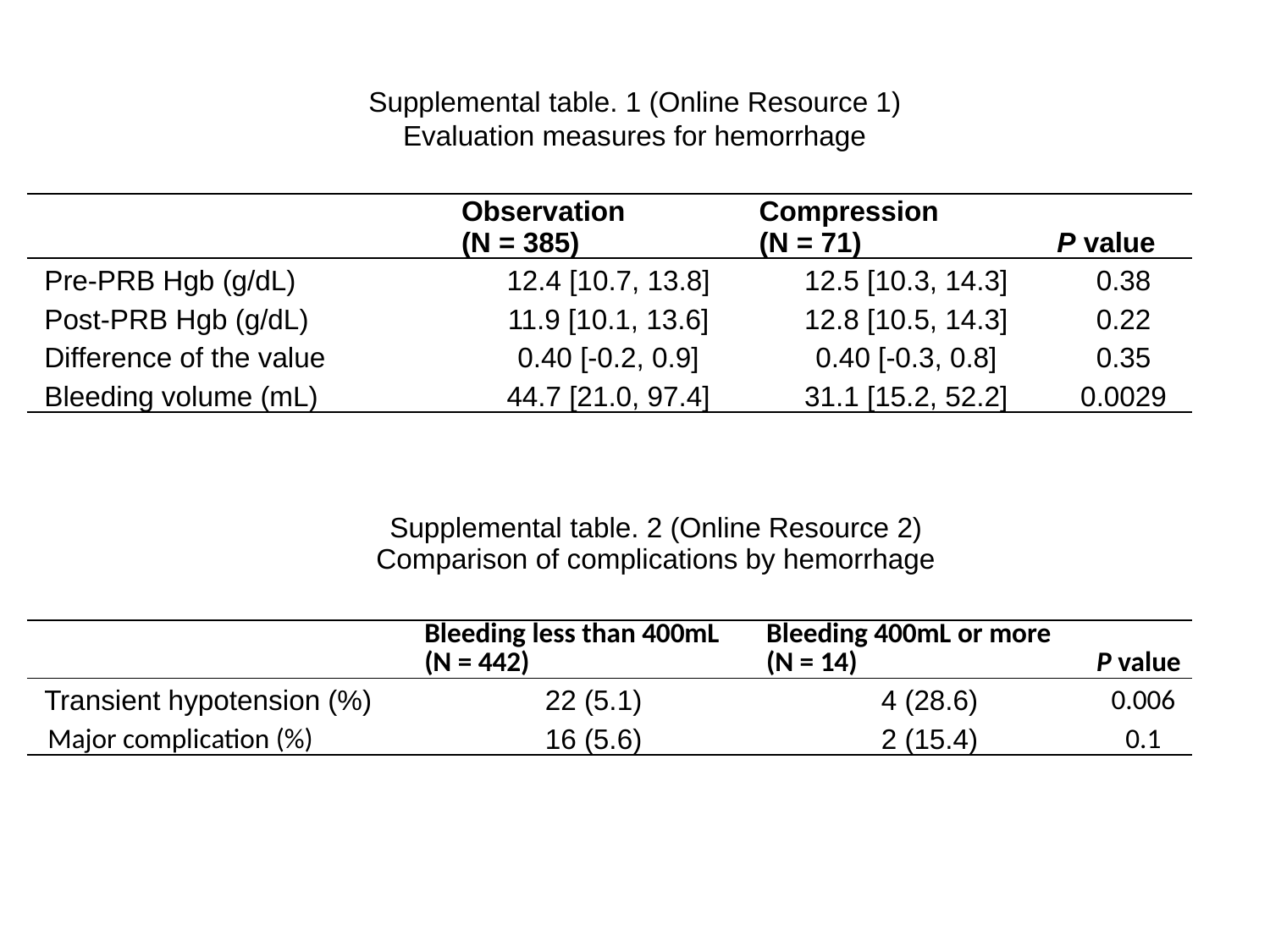

# Supplemental table. 1 (Online Resource 1) Evaluation measures for hemorrhage
| | Observation (N = 385) | Compression (N = 71) | P value |
| --- | --- | --- | --- |
| Pre-PRB Hgb (g/dL) | 12.4 [10.7, 13.8] | 12.5 [10.3, 14.3] | 0.38 |
| Post-PRB Hgb (g/dL) | 11.9 [10.1, 13.6] | 12.8 [10.5, 14.3] | 0.22 |
| Difference of the value | 0.40 [-0.2, 0.9] | 0.40 [-0.3, 0.8] | 0.35 |
| Bleeding volume (mL) | 44.7 [21.0, 97.4] | 31.1 [15.2, 52.2] | 0.0029 |
Supplemental table. 2 (Online Resource 2)
Comparison of complications by hemorrhage
| | Bleeding less than 400mL (N = 442) | Bleeding 400mL or more (N = 14) | P value |
| --- | --- | --- | --- |
| Transient hypotension (%) | 22 (5.1) | 4 (28.6) | 0.006 |
| Major complication (%) | 16 (5.6) | 2 (15.4) | 0.1 |
