# Supplementary material for: Manual compression and reflex syncope in native renal biopsy
Source: Clin Exp Nephrol. 2018 Mar 14;22(5):1100–7. doi: 10.1007/s10157-018-1560-8 (PMC6154117; doi:10.1007/s10157-018-1560-8)
Supplement: Supplementary file 2 — Supplementary material 2 Comparison of complications by hemorrhage. Data are presented as number (percentage). Major complication was assessed by counting the case of blood transfusion usage, angiographic intervention, or bladder obstruction. Fisher’s exact test was used for each binominal variable to compare differences between two groups (DOCX 15 KB) [file 10157_2018_1560_MOESM2_ESM.docx]

**Title:** Manual compression and reflex syncope in native renal biopsy

**Journal Name:** *Clinical and Experimental Nephrology*

**Authors:** Yoichi Takeuchi, M.D., Ph.D., Yoshie Ojima, M.D., Saeko Kagaya, M.D., Satoshi Aoki, M.D., Ph.D., Tasuku Nagasawa, M.D., Ph.D.

**Affiliation:** Division of Nephrology, Department of Medicine, Japanese Red Cross Ishinomaki Hospital, Nishimichishita-71 Hebita, Ishinomaki, Miyagi 986-8522, Japan

**Corresponding Author:** Yoichi Takeuchi; E-mail: muu_tko@yahoo.co.jp

Online Resource 1. Evaluation measures for hemorrhage

|  | Observation (N = 385) | Compression (N = 71) | *P* value |
| --- | --- | --- | --- |
| Pre-PRB Hgb (g/dL) | 12.4 [10.7, 13.8] | 12.5 [10.3, 14.3] | 0.38 |
| Post-PRB Hgb (g/dL) | 11.9 [10.1, 13.6] | 12.8 [10.5, 14.3] | 0.22 |
| Difference of the value (g/dL) | 0.40 [-0.2, 0.9] | 0.40 [-0.3, 0.8] | 0.35 |
| Bleeding volume (mL) | 44.7 [21.0, 97.4] | 31.1 [15.2, 52.2] | 0.0029* |

Continuous variables are presented as median [25th and 75th percentile]. Mann-Whitney U test were used for these non-parametric variables. * significant difference at *P* < 0.05.

PRB, percutaneous renal biopsy; Hgb, hemoglobin.
